# Bioprospecting Antarctic Microorganisms: Investigating *Hymenobacter psoromatis* PAMC26554 for Biochemical Potential

**DOI:** 10.4014/jmb.2412.12010

**Published:** 2025-03-13

**Authors:** Hirotake Yamaguchi, Ryoichi Yamada, Jun Hyuck Lee, Tae-Jin Oh

**Affiliations:** 1 Department of Life Science and Biochemical Engineering, Graduate School, Sun Moon University, Asan 31460, Republic of Korea; 2 Research Unit of Cryogenic Novel Materials, Korea Polar Research Institute, Incheon 21990, Republic of Korea; 3 Bio Big Data-Based Chungnam Smart Clean Research Leader Training Program, Sun Moon University, Asan 31460, Republic of Korea; 4 Genome-Based BioIT Convergence Institute, Asan 31460, Republic of Korea; 5 Department of Pharmaceutical Engineering and Biotechnology, Sun Moon University, Asan 31460, Republic of Korea

**Keywords:** *Hymenobacter*, secondary metabolites, phenylacetic acid, lipase inhibition

## Abstract

Microorganisms from the genus *Hymenobacter* are known for their distinctive traits, yet their secondary metabolite (SM) production has not been thoroughly investigated. In this study, we examined the biosynthetic potential of SMs from *Hymenobacter psoromatis* PAMC26554, which was isolated from Antarctic lichen. In silico analysis identified biosynthetic gene clusters (BGCs) for terpenes, non-ribosomal peptide synthetases (NRPSs), and polyketide synthases (PKSs), indicating the strain’s potential for novel SM production. Optimization of culture conditions showed that R2A medium at 15°C supported growth. HPLC analysis revealed phenylacetic acid (PAA) as a notable compound, which was characterized by ESI-MS and NMR, marking the first isolation of PAA from the genus *Hymenobacter*. In addition, bioactivity assays indicated moderate lipase inhibition by PAA, while molecular docking studies revealed stable interactions with the enzyme, demonstrating that hydrogen bonding and π-π stacking contribute to its lipase inhibitory activity. In summary, this research highlights the genus *Hymenobacter* as a potential source for secondary metabolite discovery, with PAA exemplifying its unexplored biochemical capabilities.

## Introduction

As one of the most extreme ecosystems on Earth, the Antarctic environment is characterized by sub-zero temperatures, high ultraviolet (UV) radiation, low nutrient availability, and saline conditions. These harsh elements have led to unique microbial communities that have evolved specialized adaptations to survive extreme UV exposure and low temperatures [1]. The study of these microorganisms has garnered increasing interest in the field of natural product research, as they may possess distinctive biosynthetic pathways and produce novel compounds with potential pharmaceutical applications [2-4].

Among these extremophiles, *Hymenobacter* spp. represent a unique and underexplored genus of bacteria predominantly found in diverse environments, including soil, aquatic systems, and extreme habitats such as polar regions [5, 6]. These bacteria are particularly recognized for their distinctive red-pink pigmentation, derived from carotenoids, which have gained interest for their potential applications in biotechnology due to their antioxidant and UV-protective properties [7, 8]. Despite their prevalence in nature, research on *Hymenobacter* spp. remains limited, particularly regarding their metabolic capabilities and ecological roles in polar environments like Antarctica. The cultivation of *Hymenobacter* spp. under laboratory conditions has historically been challenging, with liquid culture methods proving difficult to establish and often requiring highly optimized conditions [9]. As a result, while *Hymenobacter* spp. are well known for their pigment production, research on their SMs is limited [8]. This lack of reports on bioactive compounds other than pigments suggests untapped potential, especially since many pigment-producing bacteria are recognized for synthesizing diverse metabolites with pharmacological and industrial significance.

Therefore, we sought in this study to explore the biosynthetic potential of *Hymenobacter psoromatis* PAMC26554, which was isolated from Antarctic lichens. To date, only genomic studies have been conducted on this strain [10, 11]. Through cultivation under optimized liquid culture conditions, we attempted to identify and characterize any predominant SMs produced by this bacterium. Phenylacetic acid (PAA), the primary compound isolated during this study, was identified through structural analyses using nuclear magnetic resonance (NMR) and liquid chromatography–mass spectrometry (LC-MS) [12-14], thus representing the first report of this metabolite in the genus *Hymenobacter*. Furthermore, bioactivity assays and molecular docking analysis indicated that this compound exhibits lipase inhibitory activity, highlighting a previously undocumented bioactivity.

## Materials and Methods

Ethyl acetate (EA), hydrochloric acid (HCL) and acetic acid were purchased from Daejung Chemicals & Metals Co., Ltd. (Korea). DMSO, p-Nitrophenyl dodecanoate, porcine pancreatic lipase (L3126), and orlistat were purchased from Sigma-Aldrich (USA). All other chemicals and solvents used were of the highest commercially available grade (ACS, HPLC grade; Thermo Fisher Scientific, Republic of Korea ).

### Bacterial Strain and Genome Mining of Biosynthetic Gene Clusters

The endosymbiotic bacterium *Hymenobacter psoromatis* PAMC26554 was obtained from the Korean Polar Research Institute (KOPRI) in Incheon, Korea. This strain was isolated from Antarctic lichen collected at Barton Peninsula, King George Island, Antarctica (62°13'S, 58°47'W). The complete genome information for this strain has been reported by the authors [10] and is available in the NCBI database under GenBank accession number NZ_CP014771.1. The biosynthetic gene clusters (BGCs) and putative BGCs encoding secondary metabolites were identified using antiSMASH bacterial version 7.0 [15] with default parameters and selection of all prediction features. In the PAA biosynthetic pathway prediction, the Kyoto Encyclopedia of Genes and Genomes (KEGG)[16] was used to analyze functionally annotated genes, including associated pathways. To verify the accuracy of gene function assignments, NCBI BLASTp [17] was utilized, providing additional support for the results obtained during the annotation process.

### Culture Condition Optimizations

Initially, a colony of the bacterial strain was cultivated on R2A agar medium (2 g/l peptone, 4 g/l yeast extract, 10 g/l starch, 18 g/l agar, pH 7.0) at 15°C for 2 weeks. To determine the optimal growth conditions for *H. psoromatis* PAMC26554, a series of liquid culture media were evaluated. Six different media were employed: R2A broth, marine broth (MB), nutrient broth (NB), brain heart infusion (BHI), tryptic soy broth (TSB), and Luria-Bertani (LB). To serve as the seed culture, the media were incubated at two temperature settings, 25°C and 15°C, with continuous shaking at 200 rpm for a period of 6 days. To induce the synthesis of SMs, 10% of this seed culture was inoculated into the production media and incubated for 8 days (200 rpm, 15°C). R2A, nutrient broth (NB), King's medium B (KB), and YPM medium (6 g/l peptone, 8 g/l yeast extract, 18 g/l mannitol) were employed as the production media.

### Extraction of Secondary Metabolites

Following fermentation, cells were removed by centrifugation and 200 × 3 ml of EA was added. The organic-aqueous mixture was vigorously shaken in a separating funnel to extract metabolites from the culture broth. The resulting organic layer was separated and collected in a separate flask, and the EA solvent was evaporated using a rotary evaporator. The obtained crude extracts were stored in a dark place at -20°C for further analysis.

### Purification and Analysis of Extracted Compounds

The dried EA extracts were reconstituted in 500 μl of methanol and analyzed using a Nexera UPLC system (Shimadzu, Japan) equipped with a C18 column. The oven temperature was set to 40°C, and compounds were detected using UV absorbance at their respective wavelengths. An HR-QTOF ESI/MS analysis was conducted using an ACQUITY UPLC System and a SYNAPT G2-S column (Waters Corp., USA). The chromatographic separation of compounds in the EA crude extract was performed using preparative HPLC on a Shimadzu LC 20 instrument (Shimadzu) with a C18 column (YMC-Pack ODS-AQ, 150 × 20 mm I.D., 10 μm). The solvent gradient was initiated with a composition of 90% H_2_O and 10% acetonitrile (ACN; HPLC grade), running at a flow rate of 10 ml/min. The proportion of ACN in the solvent was linearly increased to 100% over a period of 28 min, with a flow of 100% ACN for 3 min followed by a gradient to 10% ACN for 4 min. The purity of the isolated compounds was verified through HPLC and subsequently dissolved in acetone-d6 for further analysis. The structural elucidation of the compounds was carried out by Bruker Biospin GmbH (Germany) using NMR spectroscopy at 700 MHz. The acquired NMR spectra were analyzed using MestReNOVA version 14.0.1 software (Spain) to determine the chemical structures of the compounds.

### Lipase Inhibitory Activity Assay

The lipase inhibitory activity of PAA was evaluated following previously established methods reported in the literature, with minor adjustments made to the protocol [18-20]. Crude porcine pancreatic lipase type II (Sigma, L3126) was dissolved in a 0.1 M Tris-HCl buffer (Sigma-Aldrich) at pH 8.4, reaching a final concentration of 10 mg/ml. The mixture was gently stirred for 30 min, then centrifuged at 1,500 ×*g* for 10 min. The clear supernatant obtained after centrifugation was collected and used for further analysis. A fresh lipase solution was prepared immediately prior to use. PAA concentrations varied 0.01 to 100 mM, with orlistat as the positive control. Both the extracts and orlistat were diluted in 50% DMSO (DMSO ratio of 1:1). In Eppendorf tubes, 30 μl of PAA, 30 μl of lipase solution, and 160 μl of Tris-HCl buffer were combined. The mixture was then incubated at 37°C for 30 min with shaking at 200 rpm using a thermomixer. After the initial incubation, 30 μl of 1 mM p-nitrophenyldodecanoic acid was added, adjusting the total volume to 250 μl. A second incubation was performed at 37°C for 30 min, with shaking at 200 rpm on a thermomixer. Following the second incubation, absorbance was recorded at 405 nm using a microplate reader. Each sample was evaluated in triplicate, with results expressed as the percentage of lipase inhibition activity. The calculation was performed as follows: (optical density of control – optical density of sample) / optical density of control × 100.

### Molecular Binding Prediction

Three-dimensional docking, interaction, and binding analyses between lipase and PAA were performed using Autodock Vina version 1.1.2 (USA) along with AutoDockTools [21, 22]. The crystal structure of lipase (PDB ID: 1LPB) was retrieved in PDB format from the RCSB Protein Data Bank website (https://www.rcsb.org/). Using AutoDockTools, water molecules were eliminated, and polar hydrogens as well as Kollman charges were introduced. The final prepared structure was saved in PDBQT format. The grid box for lipase was configured according to the reference by [23], with X, Y, and Z dimensions set to 60, and centered on particular residues of interest. PAA structure was initially created using MarvinSketch 6.01 software (Hungary), then converted to 3D models and optimized to the most stable energy configuration. In addition, 2D and 3D diagrams for binding analysis were produced using Discovery Studio (USA) [24].

## Results and Discussion

### In Silico Analysis of Secondary Metabolite Biosynthesis Pathways

Microorganisms affiliated with the genus *Hymenobacter* (phylum Bacteroidetes, family Cytophagaceae) exhibit characteristics of being aerobic, nonmotile, gram-negative, and rod-shaped bacteria. In liquid media, *Hymenobacter* cells have a propensity to grow as flocs or aggregates, accompanied by the production of a viscous extracellular polymer with a slime-like consistency [25, 26]. However, no SM isolations from *Hymenobacter* spp. have been reported except for pigment compounds.

Hence, in an effort to discover novel SMs, in silico analysis was conducted to identify BGCs in *H. psoromatis* PAMC26554 (Table 1). The analysis revealed a terpene BGC with limited similarity to known genes, alongside remarkably novel NRPS and PKS gene clusters (Fig. S1). These noteworthy discoveries underscored the importance of undertaking the following experiments for the extraction and identification of SMs from *H. psoromatis* PAMC26554.

### Selection of Suitable Culture Conditions

**Fig. 1:**
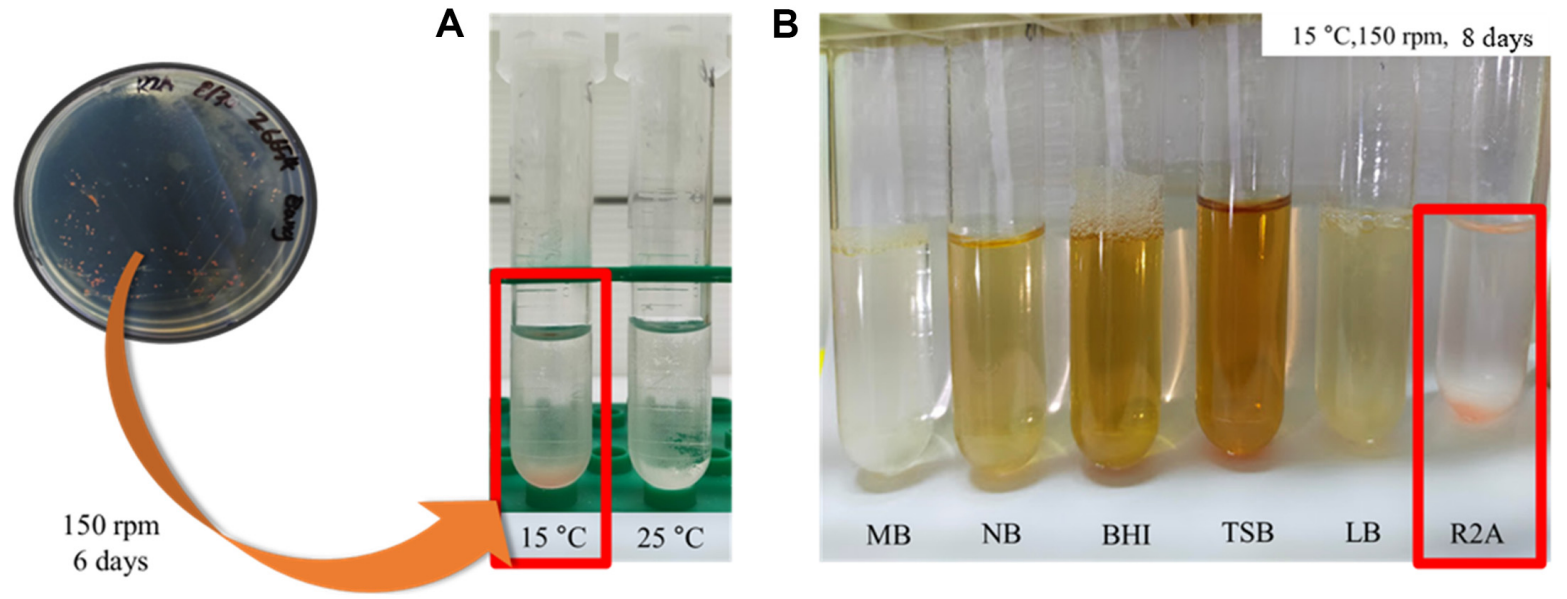
Optimization of growth conditions for *Hymenobacter psoromatis* PAMC26554. (**A**) Temperature-based growth evaluation of *Hymenobacter psoromatis* PAMC26554 cultured in R2A medium at 15°C and 25°C. (**B**) Comparison of growth across various nutrient media (Marine Broth, Nutrient Broth, BHI, TSB, LB, and R2A) at 15°C and 200 rpm.

Given the limited reports of liquid cultures for *Hymenobacter* spp., it was necessary to investigate the optimal culture conditions. Initially, we conducted temperature-based culturing experiments using R2A medium, a previously reported medium, at two different temperatures (15°C and 25°C). The results indicated that 15°C gave the most favorable growth (Fig. 1A). Different nutrient media including marine broth, nutrient broth, BHI, TSB, LB, and R2A medium were used for bacterial cultures at 15°C and 200 rpm. Notably, R2A medium showed the highest ability to support bacterial growth, although growth was also observed in NB (Fig. 1B).

**Fig. 2:**
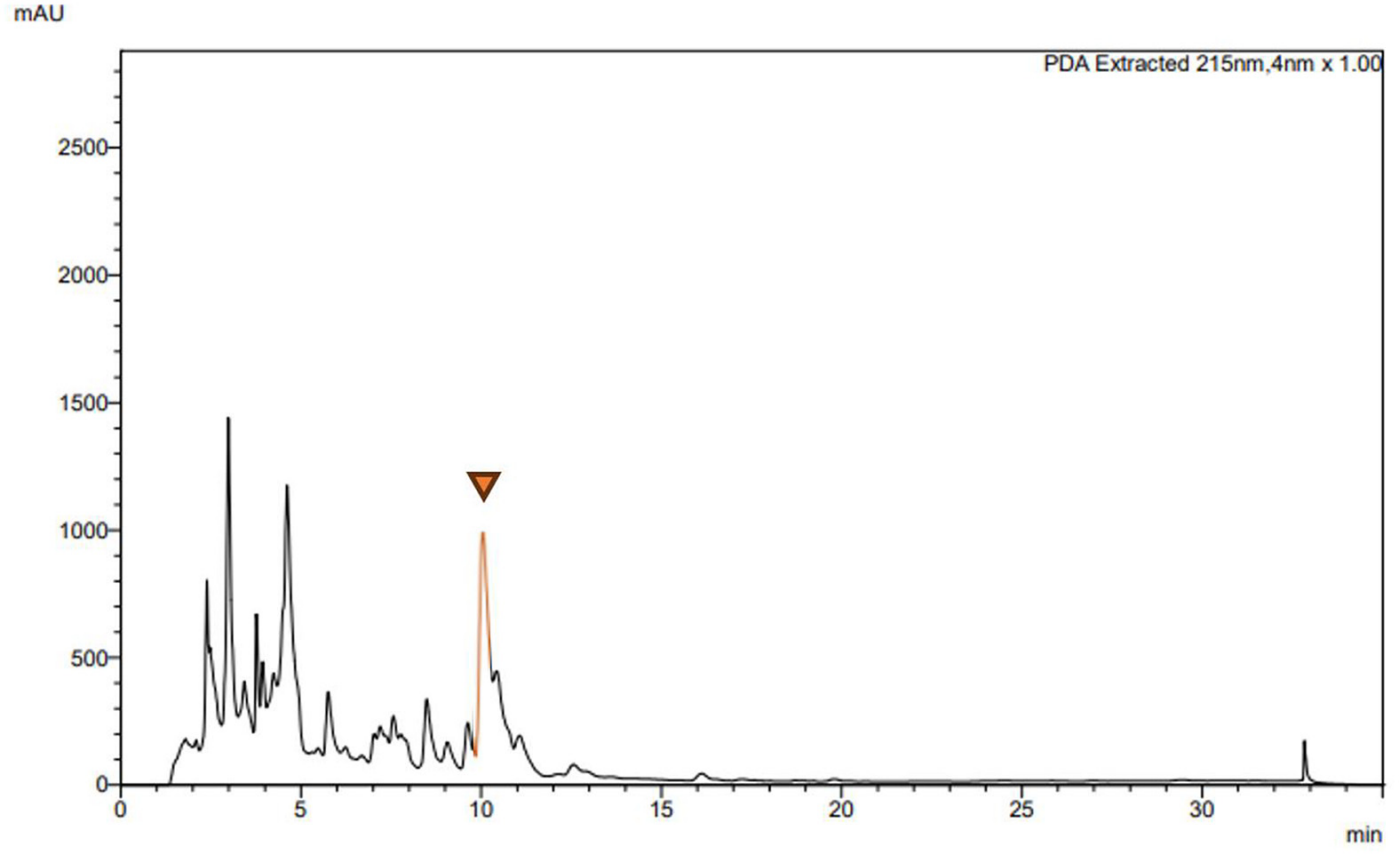
HPLC chromatogram of EA crude extract. ▼ indicates a metabolite with high production (not detected in the control medium component).

Following pre-culturing with R2A medium (6 days, 15°C, 200 rpm), the evaluation of SM production was conducted using four different media (R2A, NB, KB, and YPM) to identify the optimal medium for *H. psoromatis* PAMC26554 to produce its unique metabolites. After an incubation period of 8 days, the cell culture medium was centrifuged to remove bacterial cells, and crude extracts were obtained using EA. The crude extract obtained from the cell-free supernatant of the culture broth of *H. psoromatis* PAMC26554 was dissolved in HPLC-grade methanol and subjected to HPLC analysis. In the YPM medium culture extracts, a prominent peak with significantly higher intensity was observed at a retention time of approximately 10.1 min, which was absent in the control sample obtained from EA extraction of medium alone (Fig. 2, Fig. S2).

### Purification and Characterization of Extracted Compounds

**Fig. 3:**
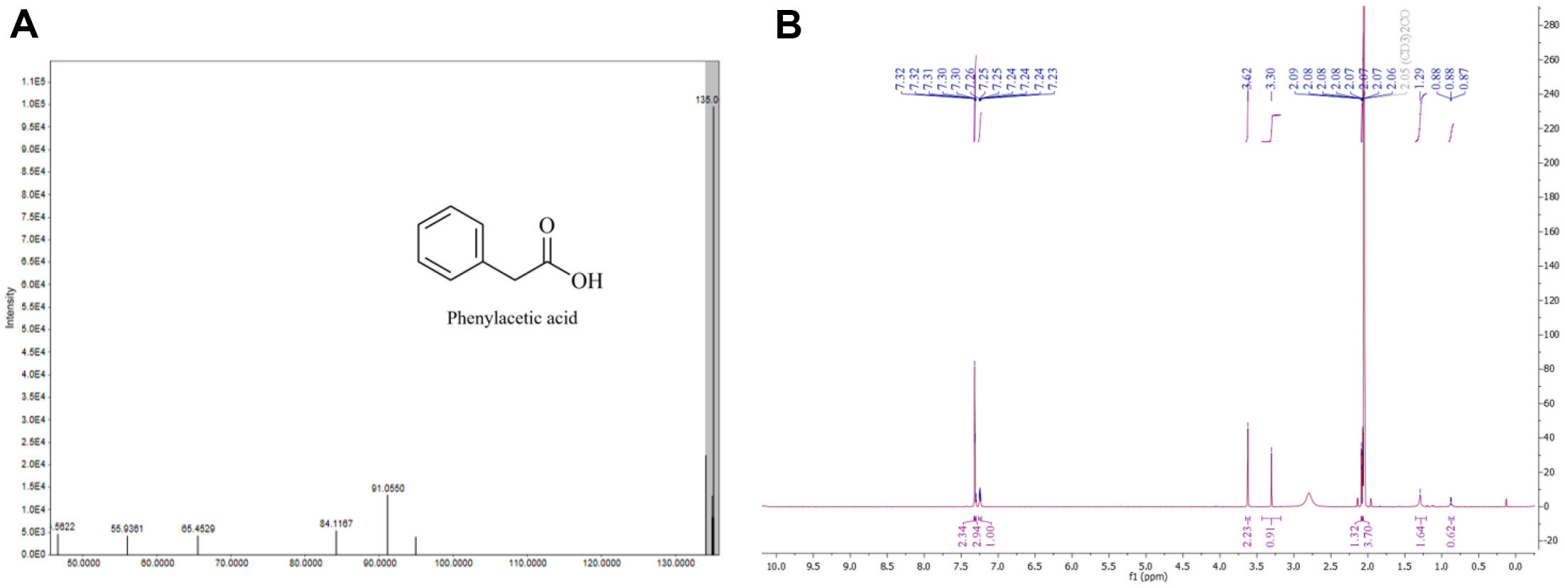
Structural analysis of an isolated compound. (**A**) Mass spectrometry detected in negative ion mode (**B**) ^1^H NMR spectra of PAA.

**Fig. 4:**
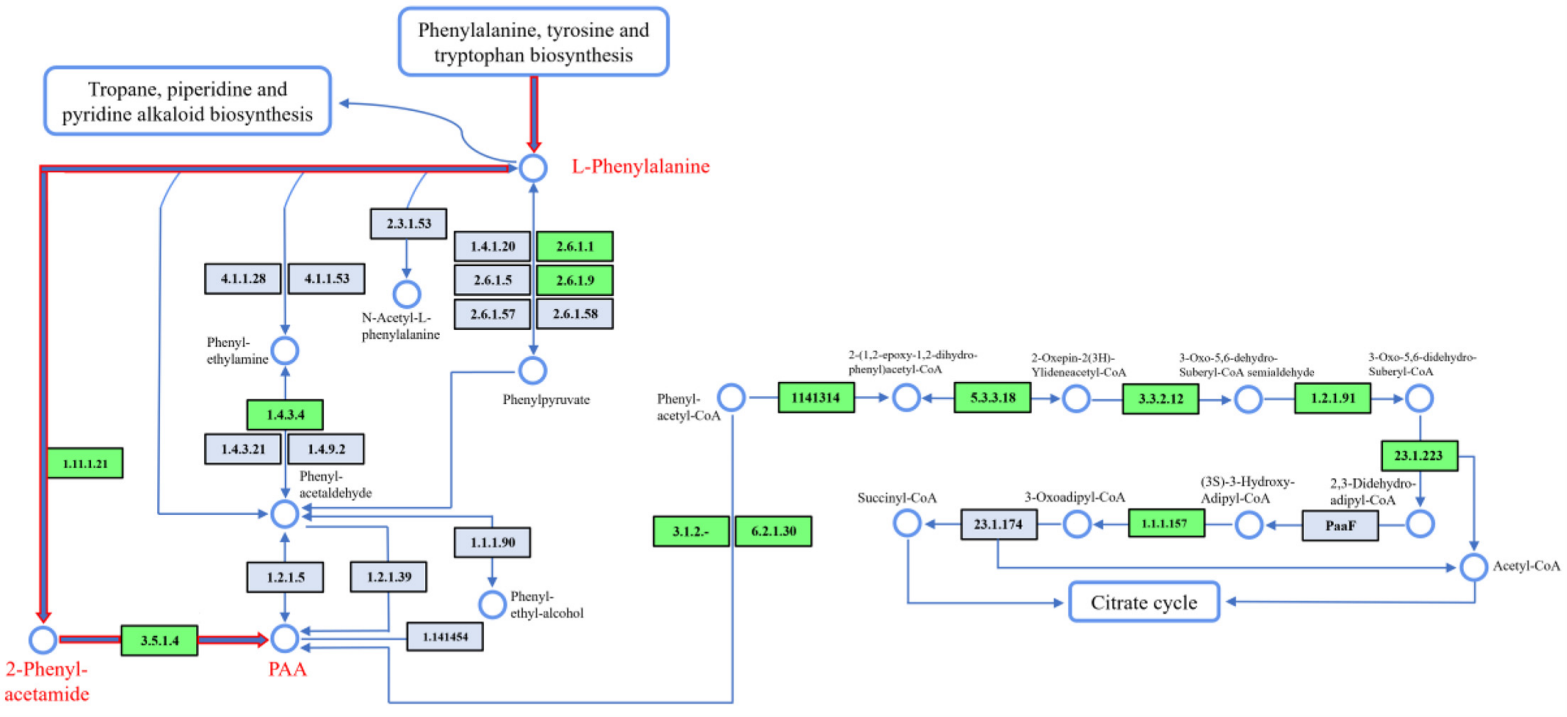
KEGG-based pathway of phenylalanine metabolism leading to PAA. The schematic pathway illustrates the enzymatic conversion of phenylalanine into phenylacetic acid highlighted in red.

The compound detected as a prominent peak was purified using preparative HPLC fractionation from the crude extract, resulting in a yield of 3.0 mg/l. ESI-MS measurements conducted in negative ion mode revealed an intense ion peak at m/z 135.04 [M-H]^-^, indicative of a molecular weight of 136 (Fig. 3A). The compound was identified as phenylacetic acid (PAA) through comparison of the data obtained from various NMR spectral analyses, including ^1^H, ^13^C NMR, and COSY (Figs. 3B, S3-S4) with reference spectra [27]. While PAA has been previously isolated from *Bacillus*, *Pseudomonas* and other bacteria [28-31], there is currently no documented evidence of its isolation from the genus *Hymenobacter*. This observation is particularly significant considering that *H. psoromatis* PAMC26554 was obtained from lichens, suggesting the potential of unexplored metabolic capabilities within this genus. The potential discovery of PAA or similar compounds in *Hymenobacter* could significantly enhance our understanding of the microbial interactions within lichens and elucidate the unique biochemical pathways that characterize these bacteria. Investigating the metabolic processes of *Hymenobacter* not only contributes to the knowledge of the genus's metabolic diversity but also underscores the ecological significance of these microorganisms in extreme environments [32]. A KEGG-based analysis has identified pathways involved in phenylalanine metabolism [33, 34], where phenylalanine is converted by catalase-peroxidase (EC 1.11.1.21) into 2-phenylacetamide. This compound is then hydrolyzed by amidase (EC 3.5.1.4) to yield PAA (Fig. 4). Further research could reveal additional insights into the enzymatic mechanisms and ecological roles of *Hymenobacter* spp.

### Exploring the Bioactivities of PAA

**Fig. 5:**
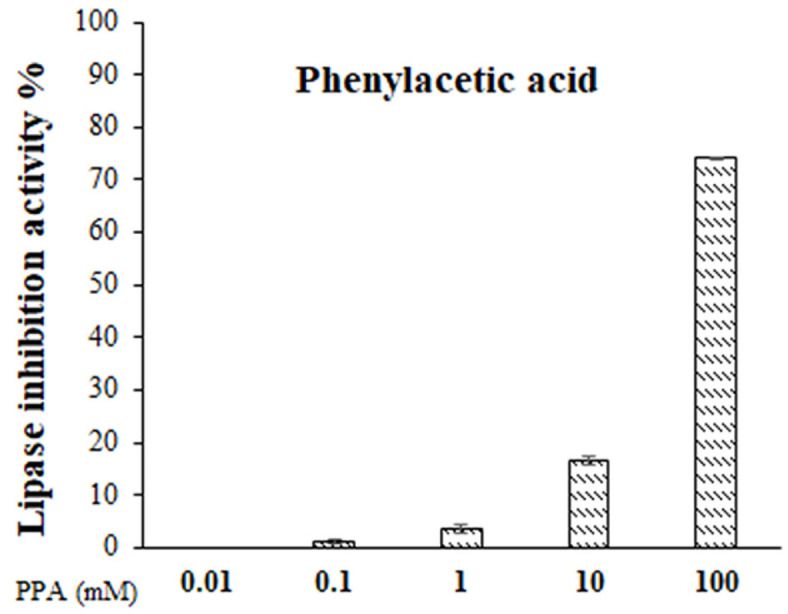
Lipase inhibitory activity of PAA. Data represent the mean ± SD of three independent experiments.

Although PAA is a well-known compound with previously reported bioactivities [35-38], this study represents its first isolation from *Hymenobacter* spp., and provides an opportunity to explore its biological activity in a novel microbial context. Despite the well-established bioactivities of PAA, its potential as a lipase inhibitor, particularly in the context of obesity treatment, has not been extensively explored in the literature. Given that lipase inhibition is a widely recognized therapeutic strategy for managing obesity, and considering the structural similarities between PAA and known lipase inhibitors [39-41], we found it both scientifically compelling and timely to investigate this aspect of its bioactivity in our study. Therefore, we investigated its potential as a lipase inhibitor. Interestingly, a slight amount of lipase inhibitory activity was detected, suggesting a previously undocumented property of PAA (Fig. 5). Lipase is an enzyme that degrades lipids, and its active site has a hydrophobic pocket. Aromatic compounds such as PAA have high hydrophobicity due to the benzene ring and may bind strongly to the hydrophobic region of lipase. In fact, simple structures with benzene rings, such as benzoic acid and cinnamic acid, have also been previously reported to have lipase-inhibiting activity [39]. These findings highlight the importance of characterizing even well-known compounds when isolated from new microbial sources, as they may display unique or unexpected activities.

### Molecular Docking Analysis of Lipase

**Fig. 6:**
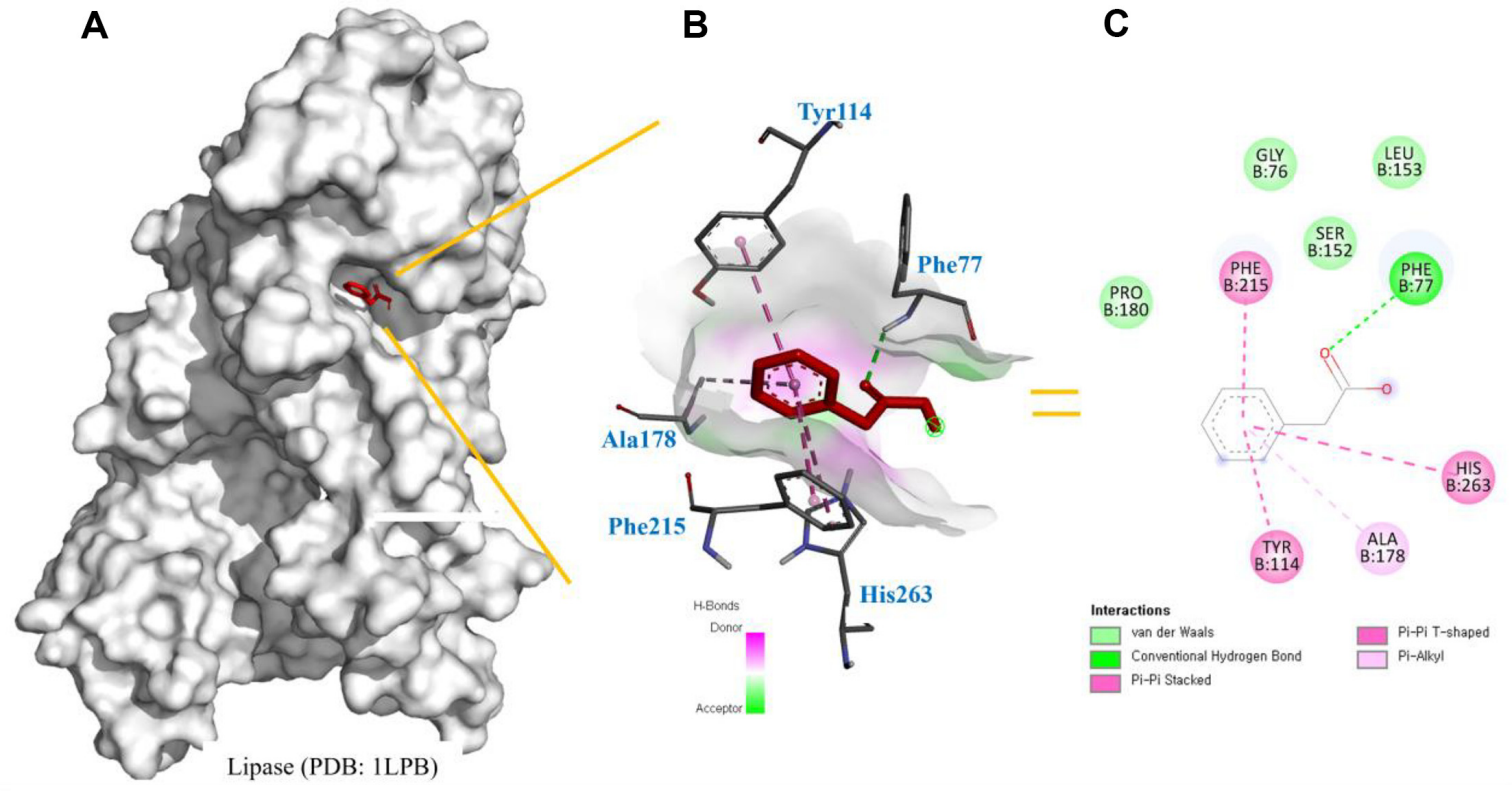
Molecular docking analysis of PAA with lipase. (**A**) Binding position of PAA (highlighted in red) on the surface of the lipase enzymés active site. (**B**) Interaction profile showing hydrogen bonds formed between PAA and residues Ser152 and Tyr114, as well as π-π interactions with Phe77 and Phe215. (**C**) Additional weak hydrophobic interactions observed between PAA and residues Ala178, Gly76, and Pro180.

Following the results of the *in vitro* assay, further molecular docking analysis was performed. As shown in Fig. 6A, the binding position of PAA (highlighted in red) is located on the surface of the enzymés active site, and it was revealed to bind at the same site as orlistat, a known competitive inhibitor. This observation further strengthens the understanding of the inhibitory potential of the compound and provides additional evidence for a competitive inhibition mechanism. The interaction analysis revealed that PAA forms hydrogen bonds with Ser152 and Tyr114, as well as π-π interactions with Phe77 and Phe215, indicating that hydrophobic bonding plays a role in stabilizing the ligand (Fig. 6B). Additionally, residues such as Ala178, Gly76, and Pro180 were found to form weak hydrophobic interactions with the ligand (Fig. 6C). These findings suggest that PAA may moderately inhibit lipase activity, offering potential as an anti-obesity drug. Stable ligand-enzyme interactions are essential for the efficacy of lipase inhibitors, as demonstrated in related studies [42, 43]. The binding profile of PAA, characterized by hydrophobic interactions and π-π stacking, indicates that enhancing its hydrophobicity and complementarity could improve its inhibitory potential. M oreover, PAA's potential dual function as both a lipase inhibitor and an antiproliferative agent merits further investigation while also presenting promising opportunities for the development of effective anti-obesity therapies.

## Conclusions

This study highlights the biosynthetic potential of *H. psoromatis* PAMC26554 isolated from Antarctic lichens and emphasizes the remarkable metabolic diversity present within microbes adapted to extreme environments. Cultivating these bacteria under optimized liquid culture conditions facilitated the isolation of phenylacetic acid (PAA), a compound reported here for the first time in the genus *Hymenobacter*. Structural analyses using nuclear magnetic resonance (NMR) and tandem mass spectrometry (MS/MS) confirmed the identity of this metabolite, while bioactivity assays demonstrated that it possesses lipase inhibitory properties. The detection of lipase inhibition broadens the biotechnological potential of microbial products from Antarctic environments, particularly within pharmaceutical research contexts. These findings illustrate the untapped reservoir of bioactive compounds within polar microbial communities and support the significance of exploring extremophiles as sources of innovative natural products. Overall, our study not only contributes to a deeper understanding of microbial adaptation and secondary metabolism in extreme environments, but also provides promising leads for future applications in drug discovery and biotechnology.

## Supplemental Materials

Supplementary data for this paper are available on-line only at http://jmb.or.kr.



## Figures and Tables

**Table 1:** Putative BGCs identified by antiSMASH in the genome of *Hymenobacter psoromatis* PAMC26554.

No.	Type	From	To	Most similar BGC (similarity %)	Reference strains
1	Terpene	2,213,495	2,233,264	Carotenoid (71%)	*Algoriphagus* sp. KK10202C
2	Terpene, T3PKS	3,173,687	3,215,631	-	-
3	NRPS, T1PKS	3,852,875	3,903,593	-	-
4	NAPAA	4,849,206	4,883,720	-	-
