# Supplementary material for: Bioprospecting Antarctic Microorganisms: Investigating Hymenobacter psoromatis PAMC26554 for Biochemical Potential
Source: J Microbiol Biotechnol. 2025 Mar 13;35:e2412010. doi: 10.4014/jmb.2412.12010 (PMC11925752; doi:10.4014/jmb.2412.12010)
Supplement: Supplementary file 1 [file jmb-35-e2412010-supple.pdf]

J. Microbiol. Biotechnol. 2025. 35: e2412010
https://doi.org/10.4014/jmb.2412.12010

Supplementary Figures

Bioprospecting Antarctic Microorganisms: Investigating *Hymenobacter psoromatis* PAMC26554 for Biochemical Potential

Hirotake Yamaguchi[1†], Ryoichi Yamada[1†], Jun Hyuck Lee[2], and Tae-Jin Oh[1,3,4,5]*

[1]Department of Life Science and Biochemical Engineering, Graduate School, SunMoon University, Asan 31460, Republic of Korea

[2]Research Unit of Cryogenic Novel Materials, Korea Polar Research Institute, Incheon 21990, Republic of Korea

[3]Bio Big Data-based Chungnam Smart Clean Research Leader Training Program, SunMoon University, Asan 31460, Republic of Korea

[4]Genome-based BioIT Convergence Institute, Asan 31460, Republic of Korea

[5]Department of Pharmaceutical Engineering and Biotechnology, SunMoon University, Asan 31460, Republic of Korea

[†]These authors contributed equally to this work

***Correspondence**

Prof. Tae-Jin Oh, E-mail: tjoh3782@sunmoon.ac.kr

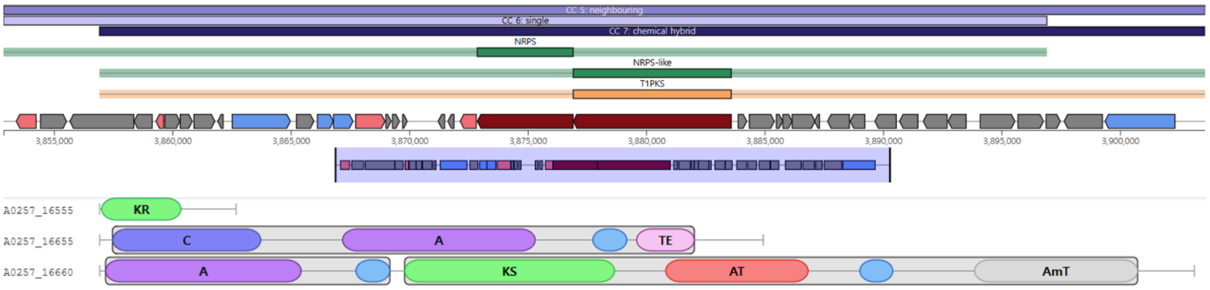

Fig. S1. The NRPS-T1PKS-NRPS-like biosynthetic gene cluster in *Hymenobacter psoromatis* PAMC26554 is illustrated. The graphical output generated by antiSMASH for region 3 is displayed, including the following components: KR (ketoreductase), C (condensation domain), A (adenylation domain), TE (thioesterase), AT (acyltransferase), and AmT (Aminotran_3). The carrier protein domains—which comprise the acyl carrier protein domain for PKS and the peptidyl carrier protein domain for NRPS—are represented by light blue ovals.

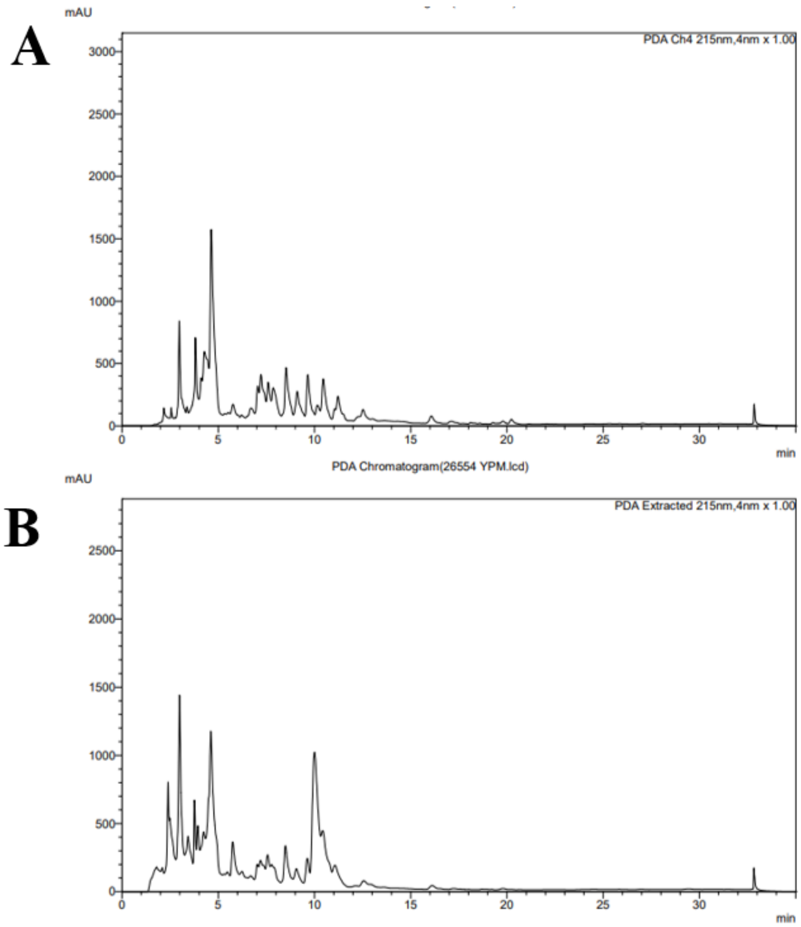

Fig. S2. **Comparison of chromatograms of EA extracts of YPM medium only (A) and EA extracts of PAMC26554 (B).**

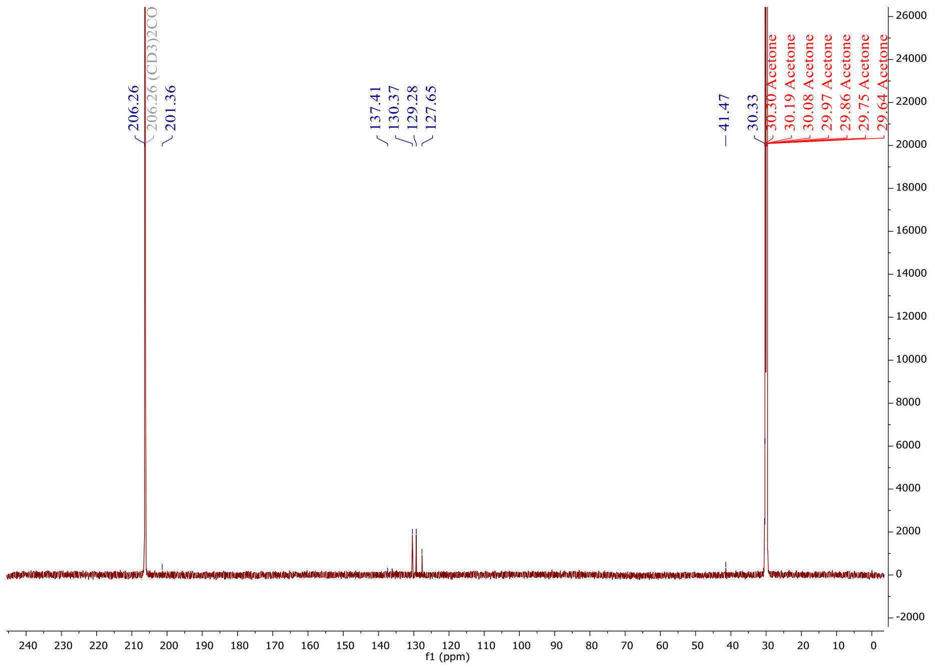

Fig. S3. **^{13}C NMR spectra of PAA.**

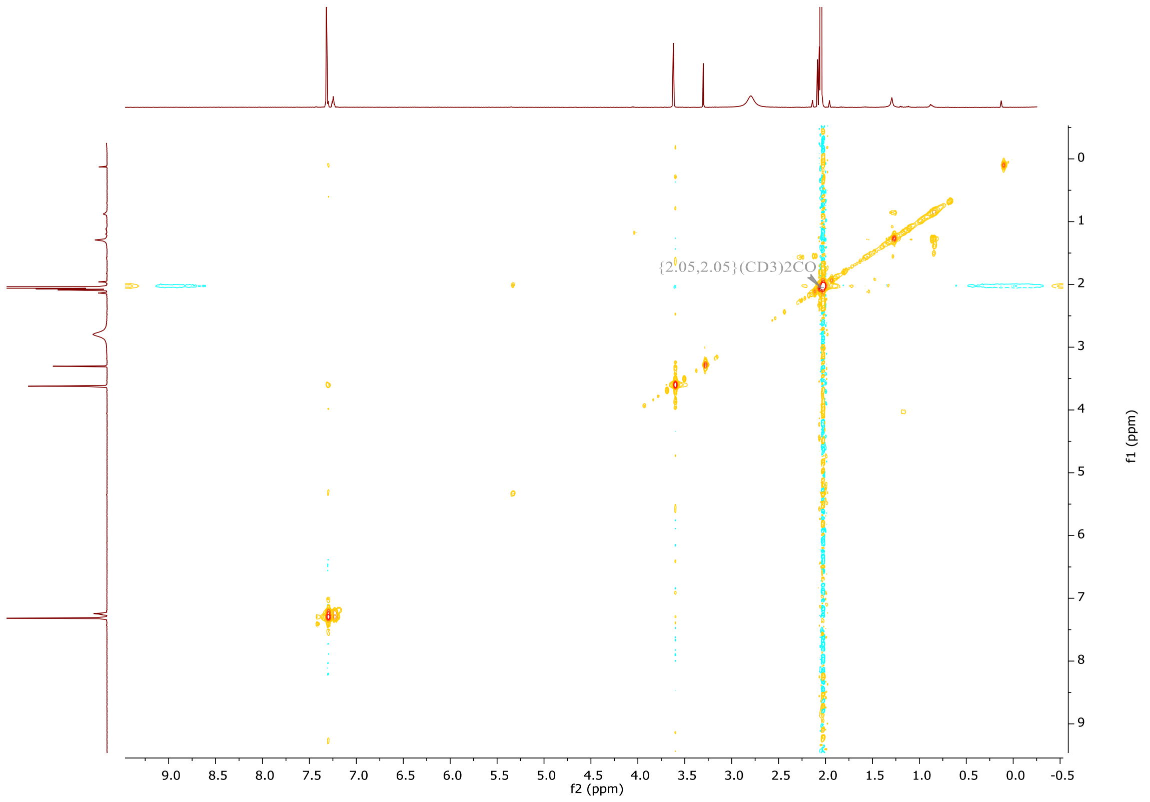

Fig. S4. **COSY spectra of PAA.**
